# Dietary inulin supplementation in early gestation regulates uterine fluid exosomes and angiogenesis to improve embryo implantation in sows

**DOI:** 10.1186/s40104-025-01247-0

**Published:** 2025-08-05

**Authors:** Junlei Chang, Xujing Pan, Wenyan Wei, Xuemei Jiang, Lianqiang Che, Yan Lin, Yong Zhuo, Bin Feng, Lun Hua, Jian Li, Jianping Wang, Mengmeng Sun, Xilun Zhao, Ruinan Zhang, De Wu, Shengyu Xu

**Affiliations:** 1 Animal Disease-Resistance Nutrition, Ministry of Education, Ministry of Agriculture and Rural Affairs, Key Laboratory of Sichuan Province, Animal Nutrition Institute, Sichuan Agricultural University, Chengdu, Sichuan 611130 People’s Republic of China; 2 College of Science, Sichuan Agricultural University, Xin Kang Road, Yucheng District, Ya’an, 625014 People’s Republic of China

**Keywords:** Angiogenesis, Embryo implantation, Exosome, Inulin, Sow

## Abstract

**Background:**

Embryo implantation in early gestation is crucial for reproductive success, while dietary fiber plays a key role in regulating sow fertility. However, the underlying mechanisms remain unclear. This study explores the influence of dietary inulin on embryonic implantation using a sow model. Sows were fed a diet supplemented with 11 g/kg of inulin during early gestation and were slaughtered on gestation day 19 (G19). Uterine fluid exosomes (UFEs) and endometrial tissues were collected for high-throughput sequencing and for analysis of the expression of angiogenesis-related genes and proteins, respectively. Furthermore, UFEs obtained from slaughtered sows were injected into G19 sows to investigate the effects on reproduction and angiogenesis.

**Results:**

The results showed that inulin significantly increased the number of blood vessels in the endometrium and expression of the angiogenesis-related proteins MMP2 and ANGPT1 in G19 sows (*P* < 0.05). Bioinformatics analysis revealed that inulin significantly downregulated miRNAs associated with angiogenesis inhibition in UFEs, while upregulating miRNAs related to trophoblast physiological activities and regulation of the uterine fluid microenvironment (*P* < 0.05). Furthermore, intravenous injection of G19 sows with UFEs from sows fed a diet containing inulin had significantly promoted vascular formation in the endometrium and embryos, and increased the number of live embryos on gestation day 28 (G28) (*P* < 0.05). Additionally, the mRNA expression levels of *MMP2*, *ANGPT1*, and *VEGF* in the placentas of sows were significantly elevated on G28 and at farrowing in the UFEs injection group (*P* < 0.05).

**Conclusion:**

Dietary supplementation with inulin during early gestation in sows promoted embryo implantation by regulating angiogenesis at the maternal–fetal interface through the modulation of miRNA expression in UFEs. These findings provide a theoretical reference for the application of dietary fiber in sow nutrition.

**Supplementary Information:**

The online version contains supplementary material available at 10.1186/s40104-025-01247-0.

## Introduction

Successful embryo implantation is essential for gestation maintenance. However, embryo implantation failure remains a major challenge with both animal reproduction and human clinical practice [1, 2]. Previous studies have shown that around 30% of embryos are lost during the implantation process in early gestation [3]. Therefore, reducing early embryonic loss during gestation in sows is a viable strategy to improve litter size and improve reproductive performance in sows.

Exosomes are vesicles with diameters of approximately 30–200 nm commonly present in blood, urine, saliva, and adipose tissue that carry cargos of various lipids, proteins, mRNA, as well as non-coding microRNA (miRNA) and small nuclear RNA (snRNA) [4–6]. During embryo implantation, uterine fluid exosomes (UFEs) act as information carriers to participate in maternal–fetal information exchange [7, 8]. Previous studies have indicated that UFEs during the embryo implantation period may be linked to physiological activities of trophoblasts and embryo implantation [9, 10]. Furthermore, exosomes derived from endometrial epithelial cells can be taken up by trophoblasts, potentially influencing reproductive performance [11]. During embryo implantation, angiogenesis at the maternal–fetal interface is essential for regulation of endometrial receptivity, placental formation, and embryonic development. Adequate endometrial thickness and a high density of blood vessels are necessary for successful embryo implantation. Insufficient vascular development may result in a range of both maternal and fetal complications during gestation, including intrauterine growth restriction, stillbirth, and miscarriage [12, 13]. Endometrial and embryonic expression levels of vascular endothelial growth factor (*VEGF*) and VEGF receptor 1 are increased during embryo implantation [14, 15]. Therefore, exosomes and angiogenesis at the maternal–fetal interface are crucial factors during embryo implantation.

As a crucial component of the sow diet, fiber significantly contributes to overall health and reproductive performance. Based on its water solubility, fiber can be divided into soluble fiber (SF) and insoluble fiber (ISF) [16]. Our research group previously reported that modulating the ratio of insoluble to soluble fiber in the sow diet during gestation can improve the uniformity and survival rate of newborn piglets [17, 18]. Inulin is a soluble fiber that can be used to adjust the ratio of insoluble to soluble fiber in the sow diet. Numerous studies have shown that inulin can improve the reproductive performance and promote growth and development of offspring. For example, inulin was reported to enhance nutritional transport in the rat placenta and reduce the coefficient of variation of fetal body weight [19, 20]. In addition, dietary inulin supplementation can improve the reproductive performance of sows and enhance the growth performance of offspring [21–23]. Dietary inulin supplementation can also improve the development and maturation of reproductive organs [24–26], thereby enhancing the reproductive performance of livestock and poultry.

The relationship between maternal nutrition in early gestation and embryonic implantation and development has recently attracted much attention [27, 28]. Currently, the mechanism by which fiber improves reproductive performance is primarily attributed to the gut microbiota and its metabolites [20, 29]. However, it remains unclear whether fiber can enhance embryo implantation by modulating UFEs and angiogenesis.

Given the significant role of inulin plays in improved reproductive performance of sows, we hypothesized that dietary inulin supplementation during early gestation could promote embryonic implantation by regulating angiogenesis through exosomes. To explore this hypothesis, the sow diet was supplemented with 11 g/kg of inulin during early gestation and the effects on UFEs and angiogenesis were examined. Additionally, gestating sows were injected with exosomes isolated from the uterine fluid of inulin-treated sows to evaluate the effects on angiogenesis, embryonic implantation, and survival. This study aims to provide a theoretical foundation to optimize reproductive efficiency in animals and optimizing nutritional strategies in human reproduction.

## Materials and methods

### Animals and experimental framework

This study was conducted at the experimental base of Sichuan Agricultural University (Ya'an, China). Animal handling and procedures received approval from the Animal Experiment Management Committee at Sichuan Agricultural University (authorization number: 20244018).

Exp. 1: Twenty primiparous LY (Landrace × Yorkshire) sows with similar body condition (body weight, 144.95 ± 1.64 kg; backfat thickness, 19.98 ± 0.34 mm) were randomly assigned to either a control group (CON, *n* = 10) or an inulin group (*n* = 10). The CON was provided with a basal diet, whereas the inulin group was given the same basal diet supplemented with 11 g/kg of inulin. The composition and nutritional profile of the basal diet are provided in Supplementary Table S1. The experimental diet was fed to sows starting with the first artificial insemination, and they were subsequently slaughtered on the morning of gestation day 19 (G19).

Exp. 2: To investigate the role of exosomes in inulin regulation of sow reproduction, exosomes from uterine fluid of the inulin group sows were injected into another batch of G19 sows. The specific experimental protocol is as follows: G19 sows were randomly assigned to the exosome group (EX, *n* = 10) or normal saline group (NS, *n* = 10), fed the same basal diet as the CON in Exp. 1, and injected via the ear vein with 5 mL of exosomes or normal saline, respectively. The protein concentration of exosomes was 1.3 mg/mL which was determined by BCA kit (Beyotime, P0010). Four sows from the EX and NS groups were slaughtered on gestation day 28 (G28) and the remaining 6 sows continued to receive standard feeding until farrowing.

All sows were fed at 8:30 and 14:30 daily, with a total feed amount of 2.3 kg/d. During the entire experimental period, the sows had free access to drinking water. The temperature and humidity of the feeding environment were kept consistent.

### Sample collection and data recorded

Exp. 1: A total of 5 mL of blood was collected and transferred into a tube containing heparin sodium as an anticoagulant. Subsequently, the blood was centrifuged at 4 °C to separate the plasma, which was then stored at −20 °C for further analysis. After the sows were slaughtered, the uterus, endometrium, and embryonic tissues were collected. The endometrial samples were taken from the middle region of the uterine horns. Portions of the uterine and embryonic tissues were fixed in 4% paraformaldehyde for subsequent histological sectioning. The remaining samples were stored at −80 °C for further analysis. The uterine weight, number of embryos and number of corpora lutea on the ovaries were recorded. Uterine fluid was obtained by washing the uterine horns of the sows with phosphate-buffered saline (PBS), using 300 mL of PBS per pig, aliquoted into 50-mL centrifuge tubes and stored at −80 °C for further analysis.

Exp. 2: Blood samples were collected from sows at 2 h and 6 h following exosome injection. Embryos and endometrial tissues were obtained at slaughter. The collection of blood, embryos, uterus and endometrial tissues was conducted as described in Exp. 1. In addition, placental tissues were collected on G28 and at farrowing. The total number of embryos, number of living embryos, number of dead embryos, and number of corpora lutea were recorded. At farrowing, total number of piglets born, number of live-born piglets, litter weight at birth, and average birth weight of live piglets were recorded.

### Hormone determination

Enzyme-linked immunosorbent (ELISA) kits (Quanzhou Ruixin Biological Technology Co., Ltd., Quanzhou, China) were used to measure plasma concentrations of estradiol (E_2_, JRX712256), progesterone (PROG, JRX710606) and vascular endothelial growth factor (VEGF, RX500921P). All determination steps were carried out according to the kit instructions.

### Hematoxylin–eosin (HE), immunofluorescence and immunohistochemical analyses

Endometrial and embryonic tissue samples were fixed with 4% paraformaldehyde, then embedded in paraffin, and cut into 5 μm-thick sections, which were stained with HE to calculate the number of blood vessels per unit area of tissue [30]. CD31 immunofluorescence and alpha-smooth muscle actin (α-SMA) immunohistochemical staining of endometrial and embryonic sections were conducted as described by Hu et al. [31]. The main steps included dewaxing, antigen retrieval, antibody incubation and nuclear staining. CD31 and α-SMA were labeled with red and blue fluorophores, respectively. The fluorescence intensity of the CD31 protein and number of positive cells were quantified with Image J software (version 1.58).

### Gene expression

RT-qPCR was used to determine the expression of endometrial angiogenesis-related genes *VEGF*, Matrix metalloproteinase 2 (*MMP2*), Matrix metalloproteinase 9 (*MMP9*), Angiopoietin 1 (*ANGPT1*), Angiopoietin 2 (*ANGPT2*), Fibroblast growth factor 2 (*FGF2*), in addition to the embryonic development-related genes Octamer binding protein 4 (*OCT4*), Sex determining region Y box 2 (*SOX2*), NANOG homeobox (*NANOG*) and Insulin-like growth factor 2 (*IGF-II*), steps are as described previously [32]. Total RNA was extracted from plasma exosomes with an exoRNeasy Serum/Plasma Maxi Isolation Kit (77144, Qiagen) and miRNA expression in exosomes was determined with a miRCURY LNA miRNA PCR Starter Kit (339340, Qiagen), in accordance with the manufacturer’s instructions. The primers used to amplify the target genes are listed in Supplementary Table S2. The β-actin and U6 were used as internal reference genes for tissue genes and miRNA, respectively. The target gene's relative amount was calculated using the 2^−∆∆Ct^ method.

### Protein expression

Western blot (WB) analysis was used to quantify the expression levels of angiogenesis-related proteins in the endometrium (VEGF, ANGPT1, ANGPT2, FGF2, and MMP2), as well as proteins related to embryonic development in the embryo (OCT4, SOX2, and NANOG). In addition, the expression levels of the exosome proteins CD9, CD63, and TSG101 were determined by WB analysis as described previously [33]. The antibody brands and dilution ratios used in WB are shown in Supplementary Table S3.

### Extraction and characterization of UFEs

**Fig. 1:**
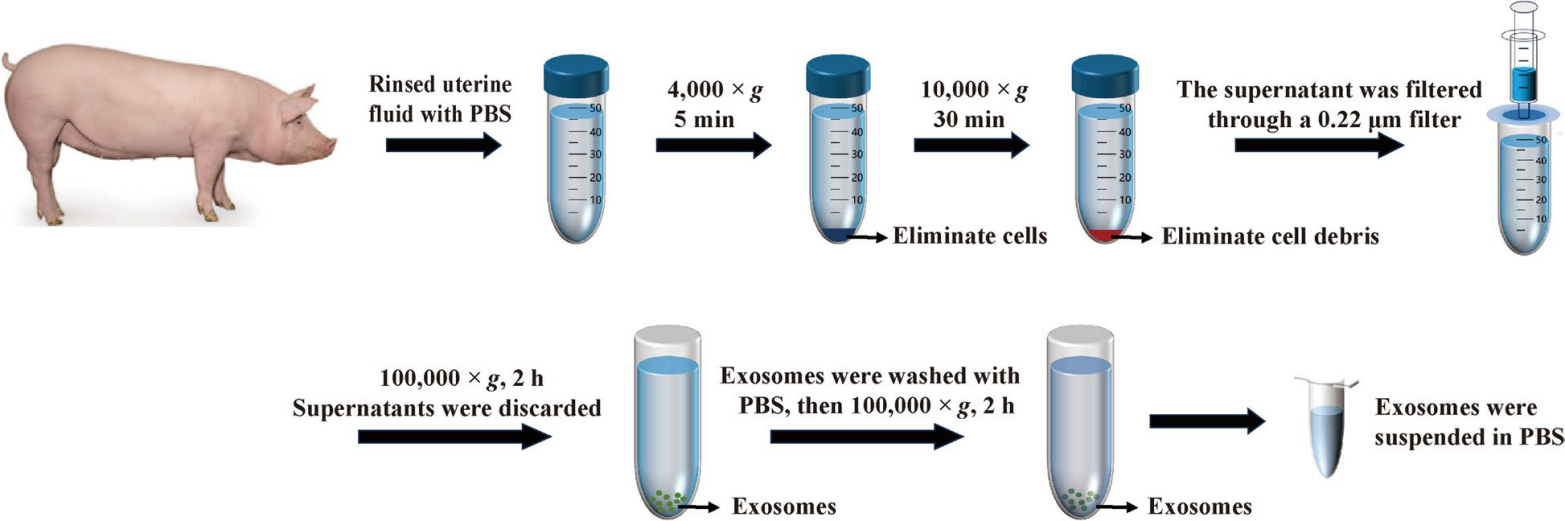
Ultracentrifugation steps of UFEs

The extraction of exosomes using ultracentrifugation method (Fig. 1). The steps of ultracentrifugation were as follows: first, centrifuged at 4 °C, 4,000 × *g* for 5 min to remove cells and tissue debris, then centrifuged at 4 °C, 10,000 × *g* for 30 min to remove smaller tissue and cell debris, and filtered the supernatant with a 0.22-μm filter membrane, collected the filtrate. The filtrate was ultracentrifuged at 100,000 × *g*, 4 °C for 120 min using a SW 32 Ti rotor (Beckman, USA) twice to collect UFEs, which were suspended in PBS and stored at −80 °C until analysis.

The characterization of exosomes was performed according to previous studies [9, 34]. Transmission electron microscope (TEM): first, dropped 10 μL of exosomes suspension onto a copper grid for precipitation for 1 min. Then, removed the remaining liquid and add 10 μL of uranyl acetate dropwise. After 1 min of precipitation, removed the remaining liquid and incubated for 3 min. Then performed imaging using a TEM (Hitachi-7700, Japan) at 100 kV. Nanoparticle tracking analysis (NTA): took 10 μL of exosome suspension and diluted to 30 μL. Then used the nanoflow cytometer (NanoFCM-N30E, China) to analyze the exosome size.

### miRNA sequencing and bioinformatic analysis of UFEs

The RNA of UFEs was extracted using exoRNeasy Serum/Plasma Maxi Isolation Kit (77144, Qiagen) following the kit instructions. Small RNA sequencing and library construction were performed by Shanghai Applied Protein Technology Co., Ltd. (APTBIO, China). The expression levels of known miRNAs were determined by mapping reads to the miRBase database, while novel miRNAs were predicted using MIREAP software (version 0.2). Differently expressed miRNAs (DEMs) were screened based on fold expression (|log_2_FoldChange| > 1) and significance of expression difference (*P* < 0.05) using DESeq (version 1.39.0). Clustering analysis of DEMs was conducted using the Pheatmap software package, while target gene prediction for DEMs was performed using Miranda (version 3.3a). Gene Ontology (GO) and Kyoto Encyclopedia of Genes and Genomes (KEGG) functional enrichment analyses of the target genes of DEMs were conducted using topGO (version 2.5) and clusterProfiler (version 4.6.0).

### Statistical analysis

SPSS 27.0 (IBM, Armonk, USA) was used to conduct tests for normality and homogeneity of variance on all data, after which the Student’s *t*-test was applied to evaluate the differences between the two groups in Exp. 1 and Exp. 2, respectively. The results are presented as the mean ± standard error of the mean (SEM), where *P* < 0.05 denotes statistically significant differences and 0.05 ≤ *P* < 0.1 indicates a significant trend.

## Results

### Effects of inulin on reproductive performance and plasma reproductive hormones in sows

**Fig. 2:**
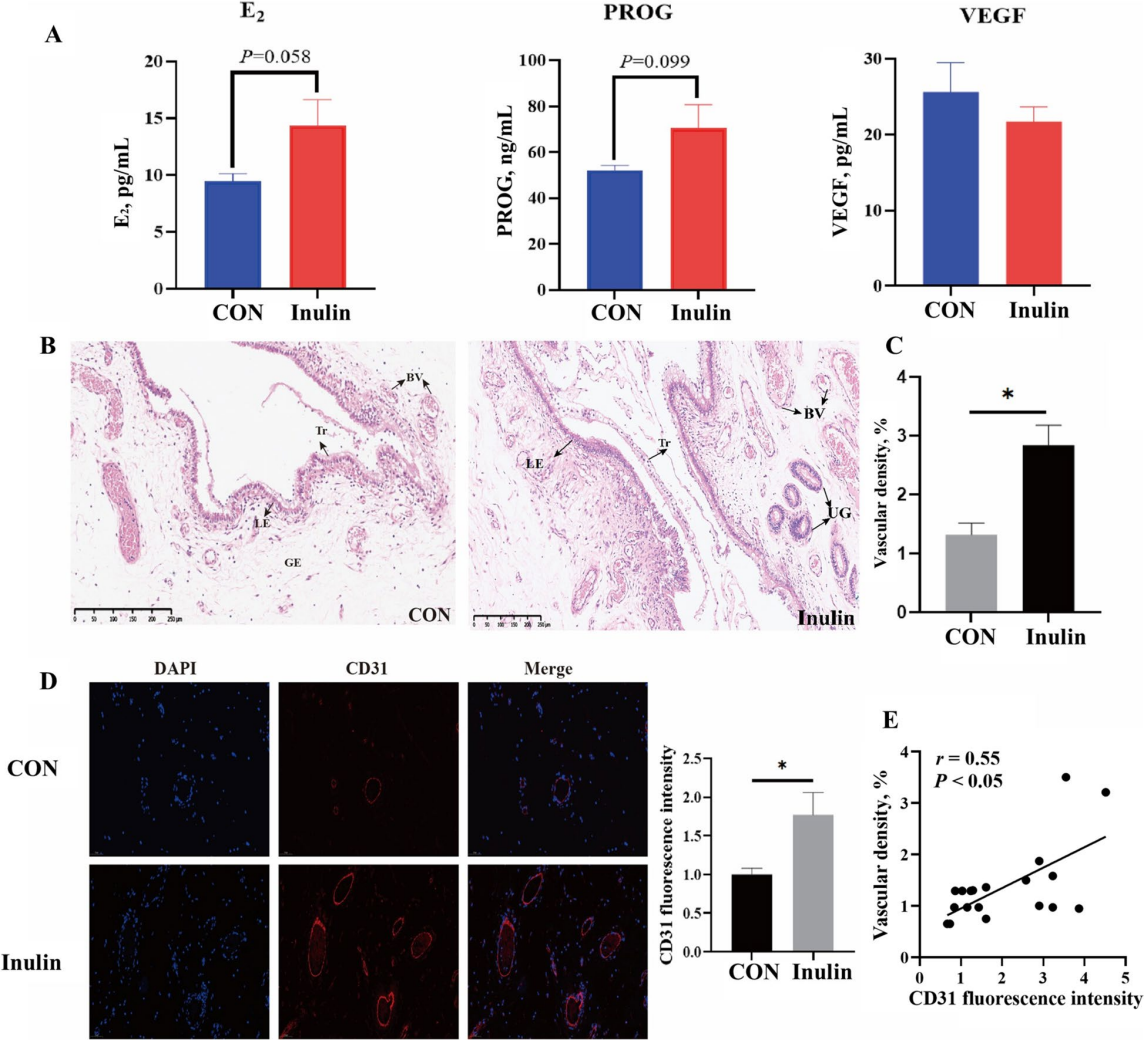
Effects of dietary inulin supplementation on hormones and endometrial angiogenesis of sows in early gestation. **A** Plasma hormone concentrations in sows. **B **and** C** Endometrial HE staining and vascular density analysis. **D** Immunofluorescence analysis of CD31 expression in the endometrium. **E** Correlation analysis between fluorescence intensity and number of blood vessels. Data are expressed as the mean ± SEM, *n* = 10. Tr: trophoblast; LE: endometrial luminal epithelial; GE: glandular epithelium; BV: blood vessel; UG: uterine gland. ^*^P < 0.05, 0.05 ≤ *P* < 0.1 indicates a trend

The results showed no significant difference in the reproductive performance of sows between the CON and Inulin (Table 1). However, Inulin tended to increase the plasma concentrations of E_2_ and PROG compared with CON (*P*_E2_ = 0.058, *P*_PROG_ = 0.099, Fig. 2A).

**Table 1:** Effects of dietary inulin supplementation on reproductive performance of sows

Items	CON	Inulin	*P*-value
Body weight at slaughter, kg	144.70 ± 2.35	145.18 ± 2.41	0.91
Backfat thickness, mm	20.97 ± 0.38	19.76 ± 0.66	0.13
Embryo number, n	25.00 ± 2.97	22.60 ± 2.87	0.58
Corpus luteum number, n	31.40 ± 4.17	30.10 ± 3.38	0.81
Relative uterine weight, g/kg	9.06 ± 0.73	7.46 ± 0.86	0.17

Data are expressed as the mean ± SEM, *n* = 10

### Effects of inulin on endometrial angiogenesis and embryonic development

**Fig. 3:**
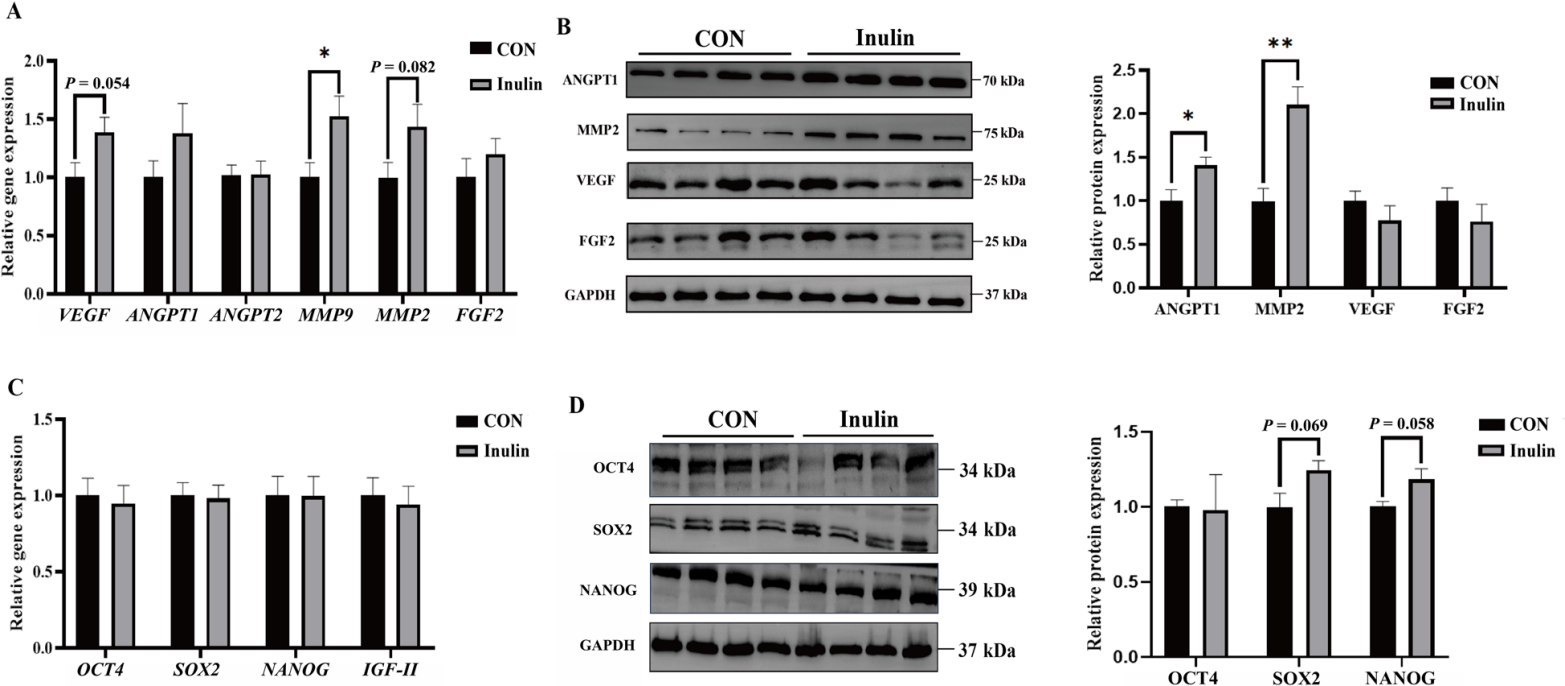
Effect of dietary inulin supplementation on endometrial angiogenesis and embryo development. **A** Expression of genes related to angiogenesis in the endometrium. **B** Expression of angiogenesis related proteins in the endometrium. **C** Expression of genes related to embryonic development in embryos. **D** Expression of embryonic development related proteins in embryos. Data are expressed as the mean ± SEM, *n* = *10*. ^*^P < 0.05, ^**^P < 0.01, 0.05 ≤ *P* < 0.1 indicates a trend

During embryo implantation, the trophoblasts adhere to the endometrium to form a double layer of epithelial cells. Endometrial biopsy showed that the porcine trophoblast layer had gradually adhered to the porcine endometrial layer (Fig. 2B), suggesting that the embryo was in the process of attachment at the time of slaughter. HE staining and CD31 immunofluorescence showed a notable increase in the density of blood vessels in the endometrium in the Inulin compared to the CON (*P* < 0.05, Fig. 2C and D), and the fluorescence intensity of the CD31 protein was positively correlated to the number of endometrial blood vessels (*r* = 0.55, *P* < 0.05, Fig. 2E). The RT-qPCR findings indicated that mRNA levels of *MMP9* in the endometrium was markedly increased in the Inulin compared to the CON (*P* < 0.05, Fig. 3A), and the *VEGF* and *MMP2* mRNA levels tended to increase (*P*_*VEGF*_ = 0.054, *P*_*MMP2*_ = 0.082, Fig. 3A). Moreover, endometrial expression of ANGPT1 and MMP2 protein levels were significantly elevated in the Inulin as compared to the CON (*P* < 0.05, Fig. 3B). Dietary inulin supplementation had no effect on the mRNA levels of the embryo development-related genes *OCT4*, *SOX2*, *NANOG*, and *IGF-II* (Fig. 3C). However, a trend towards increased protein expression of SOX2 and NANOG was observed in embryos from the Inulin compared to the CON (*P*_SOX2_ = 0.069, *P*_NANOG_ = 0.058, Fig. 3D).

### Characterization and identification of UFEs

**Fig. 4:**
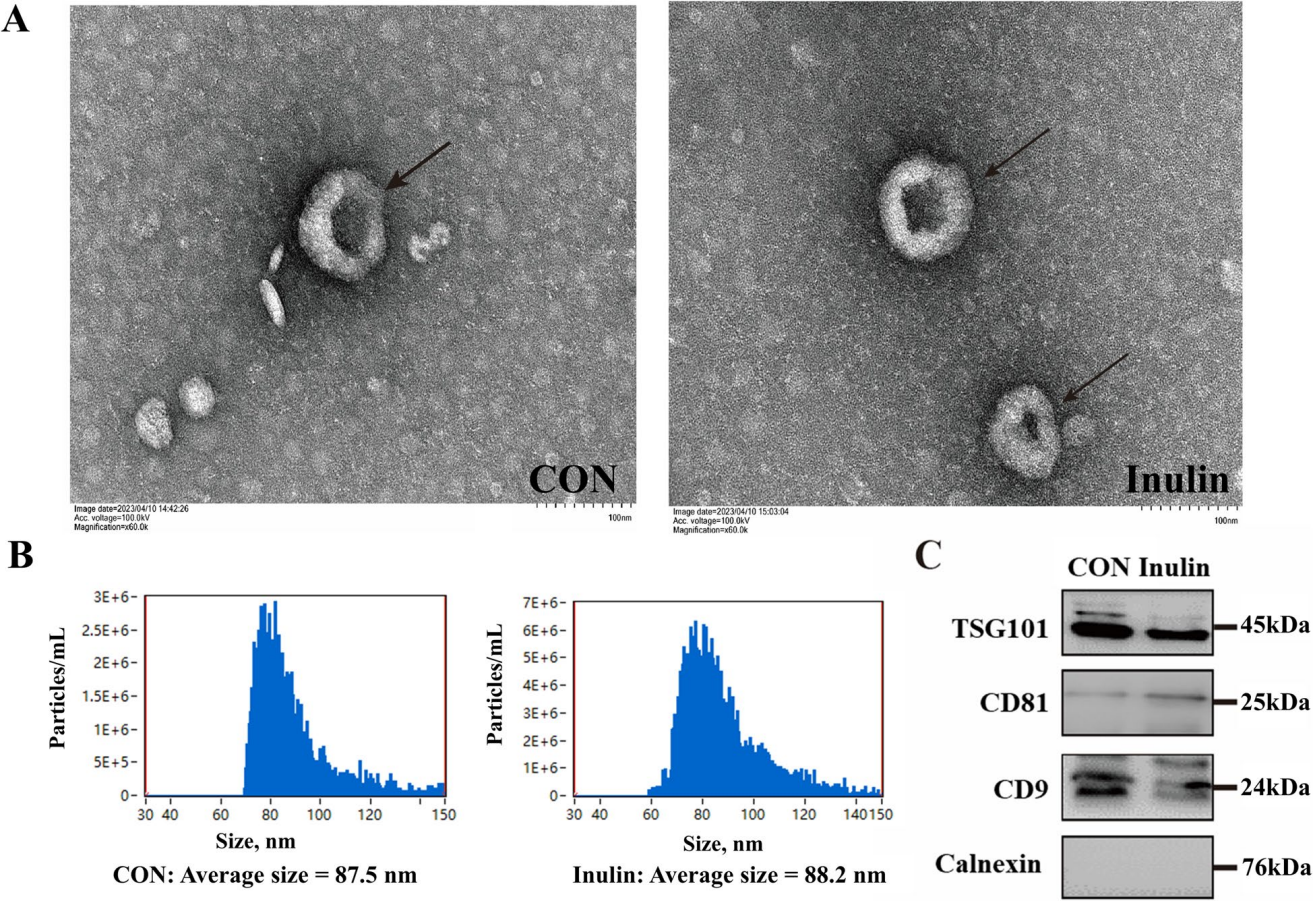
Characterization and identification of UFEs. **A** Shape of UFEs. **B** Particle size of UFEs. **C** Characteristic protein of UFEs

The UFEs in both the Inulin and CON were disc-like shaped in accordance with the exosome shape characteristics (Fig. 4A). The average exosome sizes of the CON and Inulin groups were 87.5 and 88.2 nm, respectively (Fig. 4B), in accordance with the range of exosome particle size. The WB results show that exosomes from both the CON and Inulin contained the TSG101, CD81 and CD9 proteins, but not the Calnexin protein (Fig. 4C), which is consistent with exosome characteristics.

### MicroRNA sequencing and analysis of UFEs

**Fig. 5:**
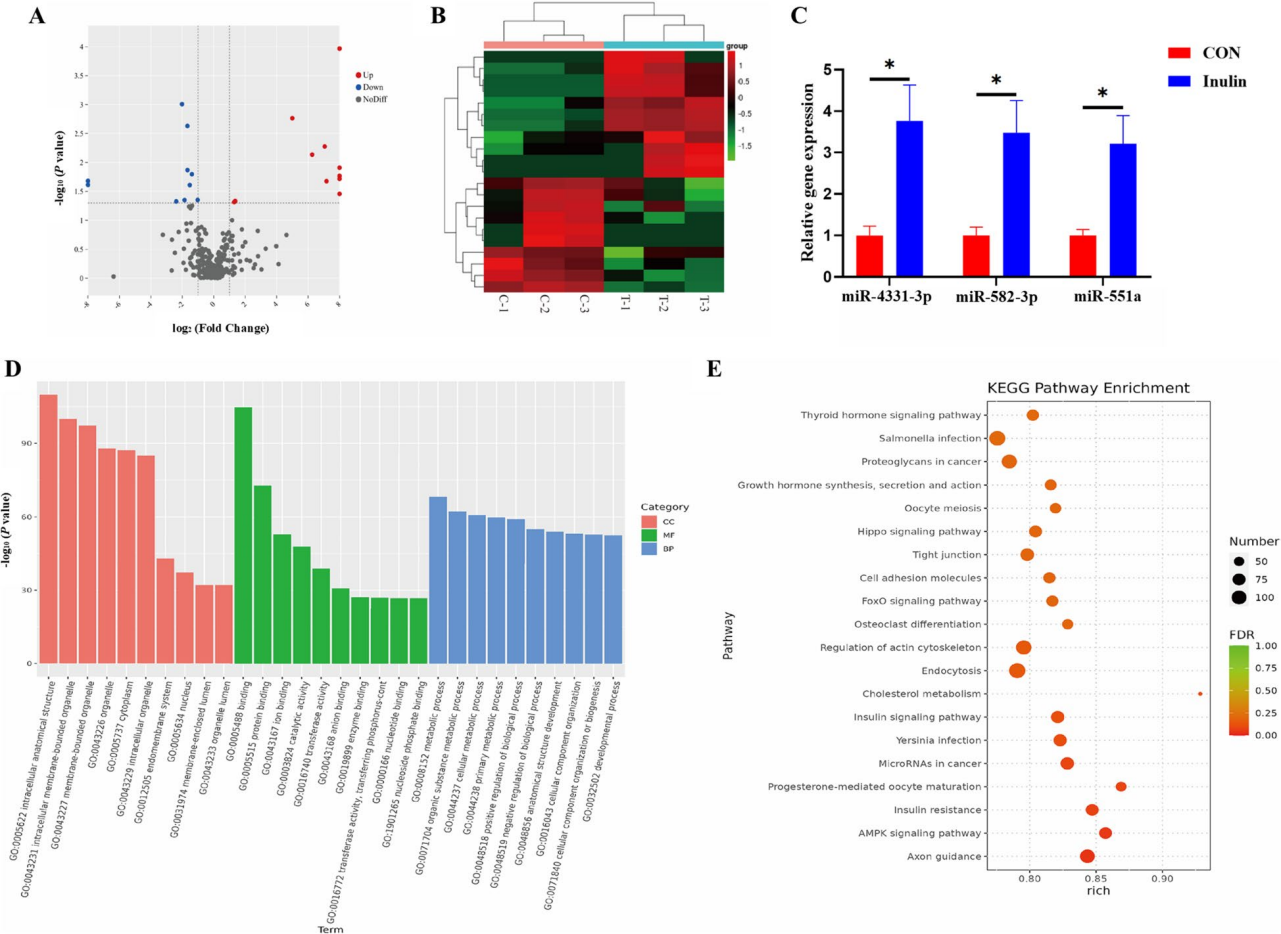
Effect of dietary inulin supplementation on the transcriptomic changes of UFEs. **A** Volcano plot of DEMs. **B** Cluster analysis of DEMs, Red represents up-regulated miRNA expression, green represents down-regulated miRNA expression. **C** The validation of the sequencing results. **D** GO (TOP 10) annotation enrichment analysis of DEMs target genes. **E** KEGG (TOP 20) annotation enrichment analysis of DEMs miRNA target genes. Rich factor refers to the ratio of the number of enriched differential miRNA target genes in the pathway to the number of annotated differential miRNA target genes. C: sows fed basal diet; T: sows fed basal diet + 11 g/kg inulin, *n* = 3. ^*^P < 0.05

The DEMs between the groups were screened according to Fold Change > 1 and *P* < 0.05. The findings indicated that Inulin, in comparison to CON (Supplementary Table S4, Fig. 5A), 11 miRNAs were up-regulated: miR-4331-3p, miR-24-2-5p, miR-664-3p, miR-493-5p, miR-2411, miR-551a, miR-582-3p, miR-2483, miR-30b-3p, miR-222, miR-421-3p; while 10 miRNAs were down-regulated: miR-183, miR-182, miR-125b, miR-1249, miR-452, miR-7137-5p, miR-326, miR-10b, miR-149, miR-34a. The DEMs cluster analysis was displayed in the form of heat map (Fig. 5B). The RT-qPCR results aligned with sequencing data, indicating the accuracy and authenticity of the sequencing results (Fig. 5C). GO and KEGG enrichment analysis of the target genes associated with DEMs was conducted. The GO enrichment results showed that the target genes of DEMs in UFEs were mainly related to protein binding, ion binding and material metabolism (Fig. 5D). The KEGG enrichment results demonstrated that the main enriched pathways of the target genes of DEMs were axon guidance, AMPK signaling pathway, insulin resistance, and cell adhesion (Fig. 5E).

### Effects of UFEs on reproductive performance and plasma hormones in sows

**Fig. 6:**
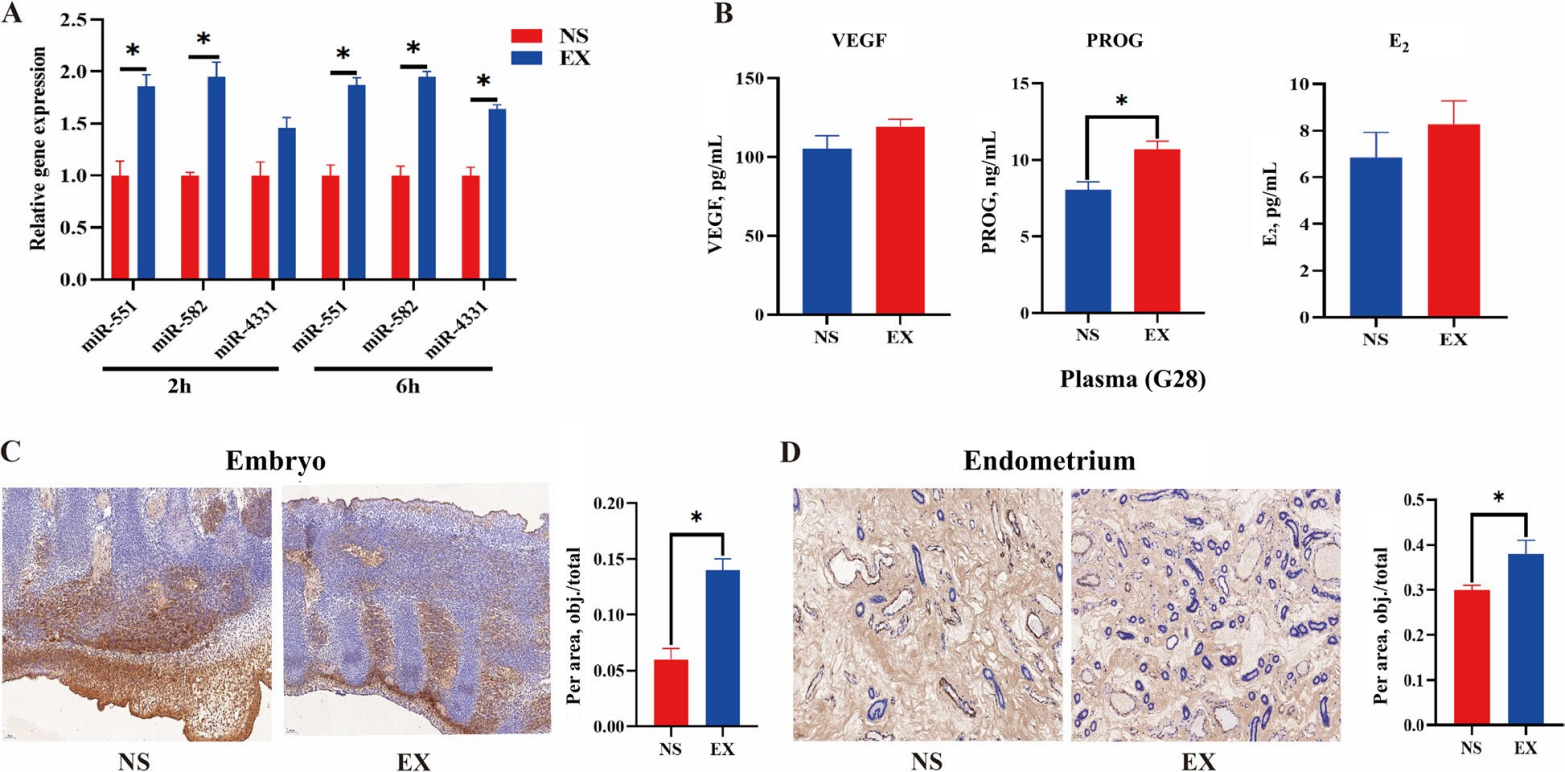
Effects of injection of UFEs on hormones and endometrial and embryonic angiogenesis. **A** Changes to miRNA in sows after UFEs injection. **B** Changes to hormones in sows on G28 after UFEs injection. **C** Embryonic angiogenesis in sows after UFEs injection. **D** Endometrial angiogenesis in sows after UFEs injection. Data are expressed as the mean ± SEM, *n* = 4. ^*^P < 0.05

Plasma miRNA expression was determined from blood collected from sows at 2 and 6 h after exosome injection. The expression levels of miR-551, miR-582, and miR-4331 in EX were higher than that in NS (*P* < 0.05, Fig. 6A), indicating that exosomes were successfully injected into sows. The findings indicated that the plasma concentration of PROG in sows on G28 was significantly increased in the EX as compared to NS (*P* < 0.05, Fig. 6B). Moreover, the injection of exosomes significantly increased the number of live embryos on G28 (*P* < 0.05, Table 2), although there was no significant effect on the reproductive performance of sows at farrowing (Table 2).

**Table 2:** Effects of maternal injection of UFEs on reproduction performance of sows

Items	NS	EX	*P*-value
Total number of embryos, n	19.50 ± 1.89	23.00 ± 1.15	0.211
Number of living embryos, n	15.50 ± 1.44^b^	22.00 ± 0.57^a^	0.015
Number of dead embryos, n	4.00 ± 1.47	1.00 ± 0.57	0.158
Number of corpora lutea, n	22.75 ± 1.44	24.00 ± 2.51	0.664
Total number of piglets born, n	15.00 ± 1.52	18.33 ± 1.20	0.161
Number of live-born piglets, n	14.33 ± 1.45	16.33 ± 0.88	0.305
Litter weight at birth, kg	18.92 ± 1.98	22.32 ± 1.12	0.210
Average birth weight of live piglets, kg	1.32 ± 0.05	1.37 ± 0.03	0.525

Data are expressed as the mean ± SEM. Slaughter data, *n* = 4. Farrowing performance data, *n* = 6

^a,b^Mean values within a row with different superscript letters were significantly different among NS and EX

### Effects of UFEs on endometrial and placental angiogenesis and embryonic development

**Fig. 7:**
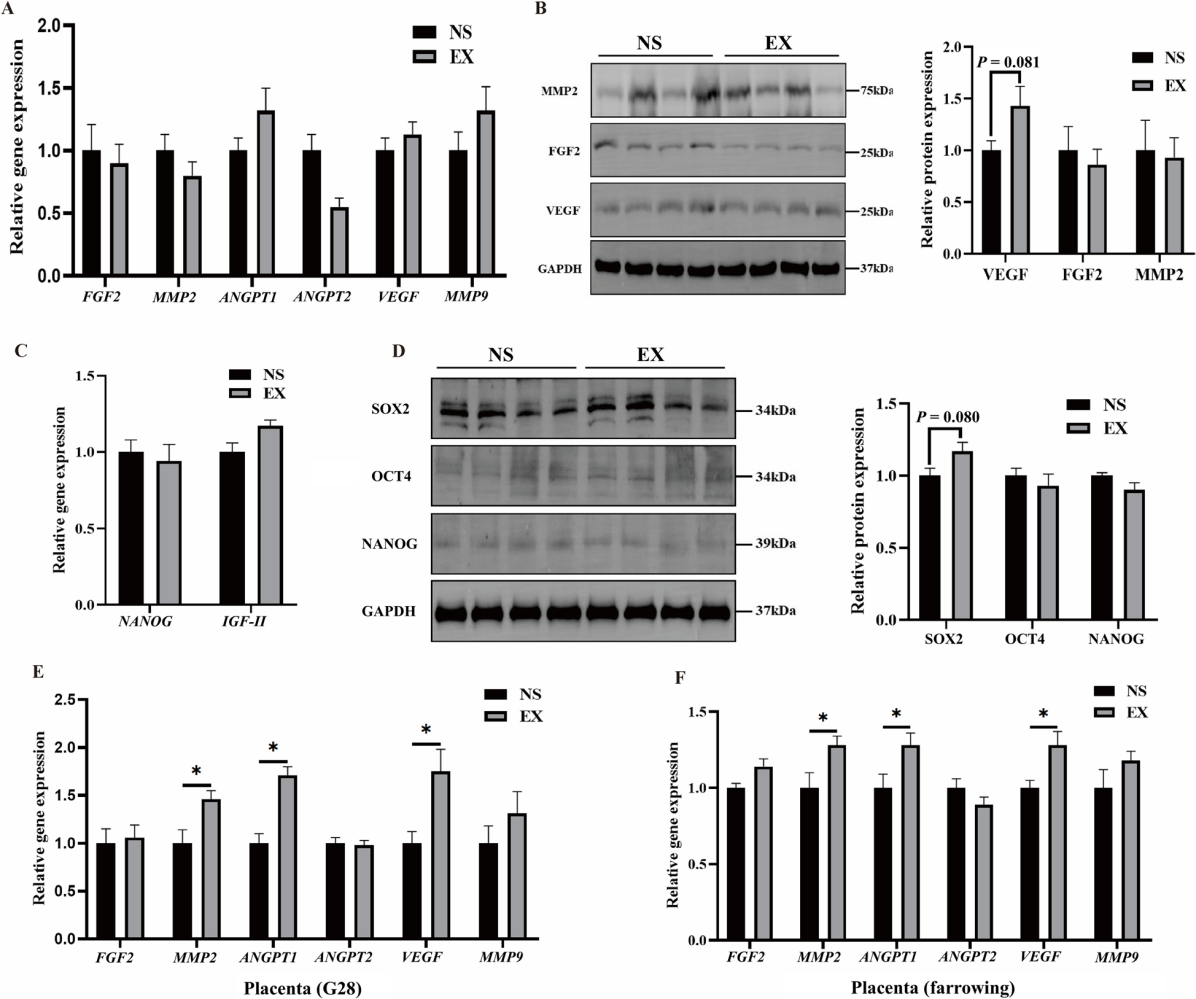
Effects of injection of UFEs on endometrial angiogenesis, embryonic development, and placental angiogenesis of sows. **A** Expression of angiogenesis-related genes in the endometrium. **B** Expression of angiogenesis-related proteins in the endometrium. **C** Expression of embryonic development-related genes in embryos. **D** Expression of embryonic development-related proteins in embryos. **E** Expression of angiogenesis-related genes in the placenta on day 28 of gestation. **F** Expression of angiogenesis-related genes in placenta on day of farrowing. Data are expressed as mean ± standard error. A–E, *n* = 4, F, *n* = 6. ^*^P < 0.05, 0.05 ≤ *P* < 0.1 indicates a trend

Immunohistochemical analysis of α-SMA showed that the number of endometrial and embryonic blood vessels was significantly increased after exosome injection (*P* < 0.05, Fig. 6C and D), while the injection of exosomes did not affect the expression of genes related to endometrial angiogenesis and embryonic development (Fig. 7A and C). However, VEGF protein expression in the endometrium and SOX2 protein expression in embryos tended to increase (*P*_VEGF_ = 0.081, *P*_SOX2_ = 0.080, Fig. 7B and D). In addition, the mRNA levels of *MMP2*, *VEGF*, and *ANGPT1* were significantly increased in the placenta on G28 and at farrowing following exosome injection (*P* < 0.05, Fig. 7E and F).

## Discussion

Dietary fiber is an indispensable component of both human and animal diets. Increasing dietary fiber levels during sow gestation can regulate the intestinal microbiota to enhance intestinal health [23, 35]. Numerous studies have shown that elevating dietary soluble fiber levels can improve the reproductive performance of sows [18, 36, 37]. Unfortunately, our findings suggest that there was no statistically significant difference in the number of embryos between the CON and Inulin, possibly because the sows were slaughtered on G19, before embryo implantation had been completed, resulting in no significant difference in embryo numbers. In addition, previous studies have been based on the reproductive performance of sows in the postpartum phase. Dietary inulin supplementation may improve sow reproductive performance by regulating the entire gestation phase, necessitating further research. Research indicates that a high-fiber diet can enhance embryo survival rates and regulate the secretion of reproductive hormones [38]. Similarly, Xue et al. [39] demonstrated that dietary inulin supplementation increased the blood concentration of E_2_ and alleviated polycystic ovary syndrome. As key steroid hormones, E_2_ and PROG are important for animal follicle development, gestation establishment and maintenance, as well as endometrial growth and development [40, 41]. Specifically, PROG promotes uterine absorption of uterine fluid during embryo implantation, thereby facilitating embryo implantation [42]. In the present study, dietary inulin supplementation tended to improve the concentration of E_2_ and PROG in sow plasma compared to CON. Therefore, it is speculated that dietary inulin supplementation may increase the concentration of E_2_ and PROG in plasma, thereby adjusting the endometrial shape and promoting the absorption of uterine fluid, which in turn facilitates embryonic implantation.

The development of uterine vasculature in early gestation is strongly correlated with embryonic development and placental angiogenesis. Abnormal uterine vascular production has been linked to infertility, abortion, intrauterine growth restriction, and preeclampsia [43, 44]. CD31 is a marker of endothelial cells, and its expression can indicate the status of angiogenesis [45]. In this study, fluorescence strength of CD31 in the endometrium was higher in the Inulin than the CON and positively correlated with the number of vascular. Uterine angiogenesis is regulated by multiple factors. VEGF is a versatile growth factor in endothelial cell growth and differentiation, and is reportedly the most crucial factor in angiogenesis [46]. The ANGPT and MMP families are also involved in endometrial angiogenesis during gestation [47, 48]. Our results indicate that dietary inulin supplementation increased protein expression of MMP9, ANGPT1 and MMP2 in endometrial, and tended to increase mRNA expression of *VEGF* and *MMP2*. Moreover, dietary inulin supplementation tended to increase *MMP9* mRNA expression in embryos. These findings indicate that dietary inulin supplementation can improve endometrial angiogenesis by regulating the expression of genes and proteins related to angiogenesis. Additionally, dietary inulin supplementation increased plasma concentrations of PROG and E_2_, which have been shown to regulate uterine angiogenesis during embryo implantation in early gestation [41, 49]. Therefore, dietary inulin supplementation may regulate angiogenesis by regulating the secretion of E_2_ and PROG, thus promoting embryo implantation. To determine whether inulin regulates early embryonic development, expression of the developmental genes *OCT4*, *NANOG*, *SOX2*, and *IGF-II* were measured in embryos. While the mRNA levels of these genes were not influenced by the dietary inulin, protein expression of SOX2 and NANOG tended to increase in the Inulin. As the earliest marker of internal cells before blastocyst formation, SOX2 collaborates with OCT4 and NANOG to establish a regulatory core that governs embryonic development [50, 51]. Previous studies indicated that NANOG and SOX2 are expressed in the inner cell mass of pig blastocysts and are also detected in the early ectoderm on gestational day 8.5, while OCT4 appears to be expressed starting on gestational day 10 [52, 53]. This experiment was conducted with sows slaughtered on G19. Consequently, it is speculated that the regulation of embryonic development by inulin requires a longer feeding period to achieve better result.

Effective communication between the embryo and the uterus is essential for the establishment of gestation [54]. Exosomes, serving as a medium for intercellular information exchange, perform a critical role in facilitating this communication [55, 56]. Numerous studies have demonstrated that the miRNAs carried by exosomes can be used as disease biomarkers, therapeutic targets, and facilitate communication between the maternal and fetal during embryo implantation [57, 58]. In the present study, UFEs were obtained by ultracentrifugation of uterine fluid. The centrifugal force and centrifugation time were referenced from the findings of Théry et al. [59] and Hu et al. [56]. The RT-qPCR results aligned with the sequencing findings, confirming the accuracy of the sequencing data. GO enrichment findings showed that the functions of the target genes of the DEMs were mainly related to cell and organic metabolism. It is well known that dietary fiber in sows can cause changes to the intestinal flora as well as nutrient digestion and metabolism. Previous studies have shown that epigenetic changes during fetal development, such as changes to DNA methylation, histone modifications, and expression of non-coding RNA, are dependent on changes to the microenvironment [60]. Maternal dietary intake influences maternal metabolism and the uterine microenvironment, thereby affecting offspring development [61, 62]. Accordingly, dietary fiber may change the uterine microenvironment via the digestive metabolism of sows, thereby regulating miRNA expression and subsequent embryo implantation. KEGG enrichment results showed that the target genes of DEMs were related to the AMPK pathway and cell adhesion. Previous research has indicated that the AMPK signaling pathway is closely linked to physiological activities such as the proliferation, differentiation, and migration of trophoblasts, and decreased AMPK expression will lead to changes to the morphology, growth rate, and nutrient transport of trophoblasts [63, 64]. Therefore, dietary ISF/SF may regulate the physiological functions of trophoblasts through miRNA in exosomes, thereby regulating embryo implantation.

Several of the downregulated miRNAs are associated with angiogenesis suppression, such as miR-182 and miR-183, which have been shown to inhibit vascular mimicry [65]. Notably, miR-125b not only inhibits angiogenesis but also increases the risk of gestation loss when overexpressed [66, 67]. Furthermore, miR-34a-3p, miR-149-5p, and miR-326-3p suppress angiogenesis by inhibiting the proliferation and invasion of vascular smooth muscle cells [68–70]. We speculate that the significant downregulation of these miRNAs may be an important factor in the improved angiogenesis in the Inulin. Therefore, to further explore the function of exosome miRNA in fiber regulation of embryo implantation, gestating sows in the embryo implantation phase were injected with exosomes obtained from sows in the Inulin. Proteins and nucleic acids carried by exosomes can serve as natural signaling molecules [71]. Because of the natural, non-toxic and biodegradable properties, exosomes have been used as carriers of vaccines or chemotherapy drugs for disease treatment [72, 73]. After exosome injection, the plasma levels of miR-551, miR-582, and miR-4331 were significantly higher at 2 h and 6 h post-injection in the EX compared to the NS, thereby confirming the successful injection of exosomes into the sows. These findings indicate that the number of living embryos in early gestation was increased, although there was no differences in the number of piglets at delivery, which may be due to the loss of embryos due to environmental and nutritional factors during mid and late gestation. In addition, since there were only four sows in each treatment group during the delivery stage, the number of repetitions was relatively small, and there may be individual differences in delivery results, which may also be one of the reasons for the lack of difference in piglet size. Staining of α-SMA revealed that embryonic and endometrial vascular development was significantly better in the EX than the NS. Additionally, the mRNA levels of genes related to angiogenesis were also significantly increased in the placenta of the EX. Previous studies have demonstrated that exosomes can mediate the delivery of miRNAs to target tissues, thereby promoting angiogenesis [74–76]. Moreover, exosomes have been clinically applied for targeted drug delivery to achieve therapeutic effects [77, 78]. For this reason, we speculate that inulin may regulate angiogenesis through miRNAs in exosomes, although further investigations are needed to elucidate the underlying mechanisms.

## Conclusion

Herein, the present work suggests that dietary inulin supplementation may regulate embryo implantation in early gestation in sows by improving angiogenesis at the maternal-to-fetal interface and regulating miRNA related to the physiological activities of trophoblasts and angiogenesis in the UFEs of sows in early gestation. However, further investigations are required to explore the specific mechanisms between UFEs and embryo implantation. These findings provide theoretical reference for the use and promotion of dietary fiber in early gestation of sows and future studies of early gestation loss and reproductive nutrition in humans.

## Supplementary Information

Supplementary Material 1: Table S1 Composition and nutrient level of experimental basal diet.

Supplementary Material 2: Table S2 Target gene primer sequences.

Supplementary Material 3: Table S3 Western Blot antibodies.

Supplementary Material 4: Table S4 Up-regulated and Down-regulated miRNAs in sow UFEs.

## Data Availability

The data supporting the findings of present study are available from the corresponding author upon reasonable request.

## Abbreviations

ANGPT1: Angiopoietin 1
ANGPT2: Angiopoietin 2
E_2_: Estradiol
FGF2: Fibroblast growth factor 2
IGF-II: Insulin-like growth factor 2
ISF: Insoluble fiber
MMP2: Matrix metalloproteinase 2
MMP9: Matrix metalloproteinase 9
NANOG: NANOG homeobox
OCT4: Octamer binding protein 4
PROG: Progesterone
SF: Soluble fiber
SOX2: Sex determining region Y box 2
TSG101: Tumor susceptibility gene 101
UFEs: Uterine fluid exosomes
VEGF: Vascular endothelial growth factor
