# Supplementary material for: Dietary inulin supplementation in early gestation regulates uterine fluid exosomes and angiogenesis to improve embryo implantation in sows
Source: J Anim Sci Biotechnol. 2025 Aug 5;16:111. doi: 10.1186/s40104-025-01247-0 (PMC12323120; doi:10.1186/s40104-025-01247-0)
Supplement: Supplementary file 1 — Supplementary Material 1: Table S1 Composition and nutrient level of experimental basal diet. [file 40104_2025_1247_MOESM1_ESM.docx]

| **Ingredient, %** | **Content** |
| --- | --- |
| Corn (CP7.8%) | 68.46  8.00  16.50  2.50  2.00  0.80  0.80  0.30  0.20  0.08  0.12  0.04  0.20  100  3.24  12.78  3.18  0.69  0.61  0.32  0.75  0.24  0.55  0.13 |
| Soybean meal (CP43%) |  |
| wheat bran |  |
| Fish meal (CP68%) |  |
| Soybean oil |  |
| Limestone |  |
| Dicalcium phosphate |  |
| NaCl |  |
| L-Lys HCl |  |
| L-Thr |  |
| Chloride choline (50%) |  |
| Vitamin premix^1^ |  |
| Mineral premix^2^ |  |
| Total |  |
| **Nutrient level^3^** |  |
| Digestible energy, Mcal/kg |  |
| Crude protein, % |  |
| Crude fiber, % |  |
| Ca, % |  |
| Total P, % |  |
| Available P, % |  |
| Lys, % |  |
| Met, % |  |
| Thr, % |  |
| Trp, % |  |

**Supplementary Table S1** Composition and nutrient level of experimental basal diet

^1^Per kilogram of diet provided: VD_3_ 1,920 IU; VA 9,600 IU; VK_3_ 3.84 mg; VE 80 IU; VB_1_ 1.6 mg; VB_2_ 5.76 mg; VB_6_ 2.88 mg; VB_12_ 20 μg; Pantothenic acid 20 mg; Biotin 0.38 mg; Folic acid 3.2 mg; Nicotinic acid 32 mg

^2^Per kilogram of diet provided: Fe 100 mg; Cu 12 mg; Mn 28 mg; Zn 120 mg; I 0.4 mg; Se 0.2 mg

^3^All are calculated values
