# Supplementary material for: Dietary inulin supplementation in early gestation regulates uterine fluid exosomes and angiogenesis to improve embryo implantation in sows
Source: J Anim Sci Biotechnol. 2025 Aug 5;16:111. doi: 10.1186/s40104-025-01247-0 (PMC12323120; doi:10.1186/s40104-025-01247-0)
Supplement: Supplementary file 2 — Supplementary Material 2: Table S2 Target gene primer sequences. [file 40104_2025_1247_MOESM2_ESM.docx]

Supplementary Table S2 Target gene primer sequences

| Gene | Primer sequence (5’→3’) |
| --- | --- |
| *ANGPT1* | F: ACAGAGCCACCACCAATAAC |
|  | R: GTGCAAAGGTTGACGAGATTATG |
| *ANGPT2* | F: CTGAGCTGTGATCTCGTCTTG |
|  | R: CTGAACCTGATACTGCCTCTTC |
| *FGF2* | F: ATATCTTCTCCAGGCTCCGACA |
|  | R: TGTGGCATCTCCATGTTTGT |
| *IGF-II* | F: CTGCTCGTCTTCTTGGCCTT |
|  | R: GGCCTGCTGAAGTAGAAGCC |
| *MMP2* | F: TGTTCACCATGGGTGGCAAT |
|  | R: ACTTCTTGTCGCGGTCGTAG |
| *MMP9* | F: ACGTGAAGACGCAGAAGGTGGATT  R: AAGATGTCGTGTGTGTTCAAGGGC |
| *NANOG* | F: GGTTTATGGGCCTGAAGAAA  R: GATCCATGGAGGAAGGAAGA |
| *OCT4* | F: CATTCAAACTGAGGTGCCTGC |
|  | R: ACTTAATCCCAAAGCCCTGGT |
| *SOX2* | F: CAGCTCGCAGACCTACATGA |
|  | R: GACTTGACCACTGAGCCCAT |
| *VEGF* | F: CCTCGGAGCGGAGAAAGCAT |
|  | R: TGTCACATCTGCAAGTACGTTCG |
| *β-actin* | F: AACTGGAACGGTGAAGGTGA |
|  | R: CTTTTGGAAAGGCAGGGACT |

*ANGPT1*: Angiopoietin 1; *ANGPT2*: Angiopoietin 2; *FGF2*: Fibroblast growth factor-2; *IGF-II*: Insulin-like growth factor-2; *MMP2*: Matrix metalloproteinase-2; *MMP9*: Matrix metalloproteinase-9; *NANOG*: Nanog homeobox; *OCT4*: Octamer binding protein 4; *SOX2*: Sex determining region Y box-2; *VEGF*: Vascular endothelial growth factor.
