# Supplementary material for: Dietary inulin supplementation in early gestation regulates uterine fluid exosomes and angiogenesis to improve embryo implantation in sows
Source: J Anim Sci Biotechnol. 2025 Aug 5;16:111. doi: 10.1186/s40104-025-01247-0 (PMC12323120; doi:10.1186/s40104-025-01247-0)
Supplement: Supplementary file 3 — Supplementary Material 3: Table S3 Western Blot antibodies. [file 40104_2025_1247_MOESM3_ESM.docx]

| Antibody | Species | Dilution ratio | Brand |
| --- | --- | --- | --- |
| VEGF | Rabbit | 1:1000 | Servicebio |
| FGF2 | Rabbit | 1:1000 | Servicebio |
| ANGPT1 | Rabbit | 1:1000 | Servicebio |
| MMP2 | Rabbit | 1:1000 | Servicebio |
| GAPDH | Rabbit | 1:1000 | Servicebio |
| NANOG | Rabbit | 1:1000 | Bioss |
| SOX2 | Rabbit | 1:1000 | Bioss |
| OCT-4 | Rabbit | 1:1000 | Bioss |
| CD9 | Rabbit | 1:1000 | Evlixir |
| CD81 | Rabbit | 1:1000 | Evlixir |
| TSG101 | Rabbit | 1:1000 | Abcam |

Supplementary Table S3 Western Blot antibodies
