# Supplementary material for: Dietary inulin supplementation in early gestation regulates uterine fluid exosomes and angiogenesis to improve embryo implantation in sows
Source: J Anim Sci Biotechnol. 2025 Aug 5;16:111. doi: 10.1186/s40104-025-01247-0 (PMC12323120; doi:10.1186/s40104-025-01247-0)
Supplement: Supplementary file 4 — Supplementary Material 4: Table S4 Up-regulated and Down-regulated miRNAs in sow UFEs. [file 40104_2025_1247_MOESM4_ESM.docx]

Supplementary Table S4 Up-regulated and Down-regulated miRNAs in sow UFEs

| Change | miRNA | CON  baseMean | Inulin  baseMean | log2  Fold Change | *P*-value |
| --- | --- | --- | --- | --- | --- |
| Up-regulated | miR-4331-3p | 0 | 253.28 | ∞ | <0.01 |
|  | miR-24-2-5p | 11.60 | 370.44 | 5.00 | <0.01 |
|  | miR-664-3p | 2.68 | 356.37 | 7.06 | <0.01 |
|  | miR-493-5p | 2.68 | 204.93 | 6.26 | <0.01 |
|  | miR-2411 | 0 | 116.47 | ∞ | <0.05 |
|  | miR-551a | 0 | 91.92 | ∞ | <0.05 |
|  | miR-582-3p | 0 | 90.31 | ∞ | <0.05 |
|  | miR-2483 | 0.89 | 128.59 | 7.17 | <0.05 |
|  | miR-30b-3p | 0 | 92.92 | ∞ | <0.05 |
|  | miR-222 | 2460.96 | 6335.26 | 1.36 | <0.05 |
|  | miR-421-3p | 490.38 | 1205.43 | 1.30 | <0.05 |
| Down-regulated | miR-183 | 59002.39 | 14551.43 | -2.02 | <0.01 |
|  | miR-182 | 54094.17 | 16945.58 | -1.67 | <0.01 |
|  | miR-125b | 21611.05 | 6788.81 | -1.67 | <0.05 |
|  | miR-1249 | 1310.41 | 499.63 | -1.39 | <0.05 |
|  | miR-452 | 141.53 | 0 | -∞ | <0.05 |
|  | miR-7137-5p | 137.97 | 0 | -∞ | <0.05 |
|  | miR-326 | 2316.30 | 804.40 | -1.53 | <0.05 |
|  | miR-10b | 181729.25 | 89261.73 | -1.03 | <0.05 |
|  | miR-149 | 1563.01 | 431.33 | -1.86 | <0.05 |
|  | miR-34a | 837.98 | 160.36 | -2.39 | <0.05 |

baseMean：normalized mean expression; CON: sows fed basal diet; Inulin: sows fed basal diet + 11 g/kg Inulin. *n* = 3.
